# 1804. Quality Improvement Project to improve empiric antibiotic therapy for skin and soft tissue infections using the nasal MRSA PCR

**DOI:** 10.1093/ofid/ofac492.1434

**Published:** 2022-12-15

**Authors:** Sabirah N Kasule, Simran Gupta, John Klyver, Lynn Chan, Teresa Seville

**Affiliations:** Bronx Healthcare Network, Bronx, NY, Long Island City, New York; Mayo Clinic Hospital in Phoenix, AZ, Phoenix, Arizona; Mayo Clinic Hospital in Phoenix, AZ, Phoenix, Arizona; Ronald Reagan UCLA Medical Center, Los Angeles, California; Mayo Clinic Hospital in Phoenix, AZ, Phoenix, Arizona

## Abstract

**Background:**

Large cohort studies from the Veteran’s Administration support the negative predictive value of the methicillin resistant *Staphylococcus aureus* (MRSA) nasal polymerase chain reaction (PCR) swabs for skin and soft tissue infection (SSTI). We investigated the use of the nasal MRSA PCR swab to reduce the inappropriate empiric use of vancomycin and piperacillin-tazobactam for SSTI at our institution.

**Methods:**

Between July and August 2021, we educated Hospital Internal Medicine (HIM) and Internal Medicine Residents (IMR) on the basics of SSTI management and the utility of a negative nasal MRSA PCR swab. Data on empiric use of vancomycin and piperacillin-tazobactam for SSTIs were collected 3/1/2021 to 7/1/2021 (pre-intervention) and 8/30/2021 to 12/29/2021 (post-intervention). We excluded patients who didn’t have an SSTI, weren’t managed by IMR or HIM, and whose empiric antibiotics were for another indication. To account for antibiotics started in the emergency department (ED), a patient was regarded as having received appropriate empiric antibiotics if they were de-escalated in the first 24 hours of being admitted to IMR or HIM.

**Figure:**
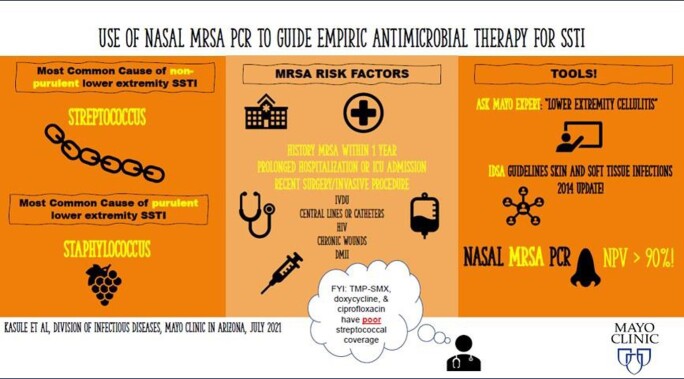

Example of education material. These fliers were distributed to resident rooms following a series of noon conferences

**Results:**

Pre-intervention there were 106 patient encounters. In 56 of these (52.8%), patients were placed on inappropriate empiric antibiotics. Post-intervention, there were 72 patient encounters. In 23of these (31.9%), patients were placed on inappropriate empiric antibiotic for an absolute risk reduction (ARR) of 20.9% with a statistically significant 95% CI (6.522 - 35.249) and a number needed to treat (NNT) of 4.78. Nasal MRSA PCR testing increased from 40.6% to 56.9%. The ARR among the HIM teams was 21.3% and was statistically significant with a 95% CI (4.24 - 38.394). Their proportion of MRSA testing increased from 41.5% to 50%. The IMR teams had an ARR of 14.8%, which was not statistically significant (95% CI, -11.339 to 40.884). Their MRSA nasal PCR testing increased from 37.5% to 72%.

**Figure:**
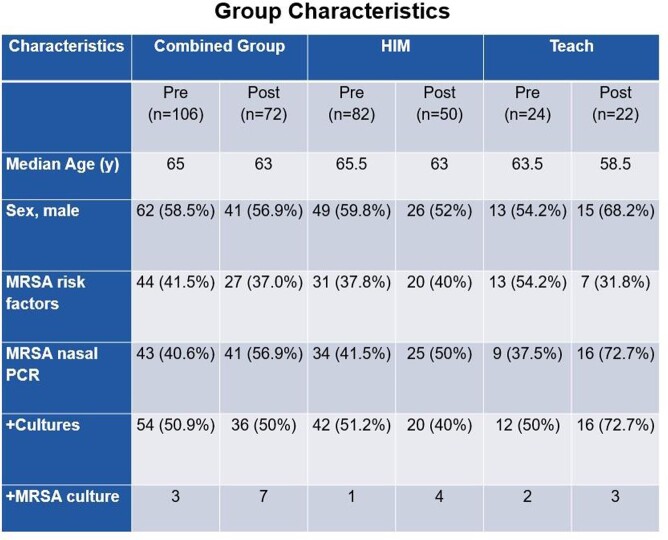

**Figure:**
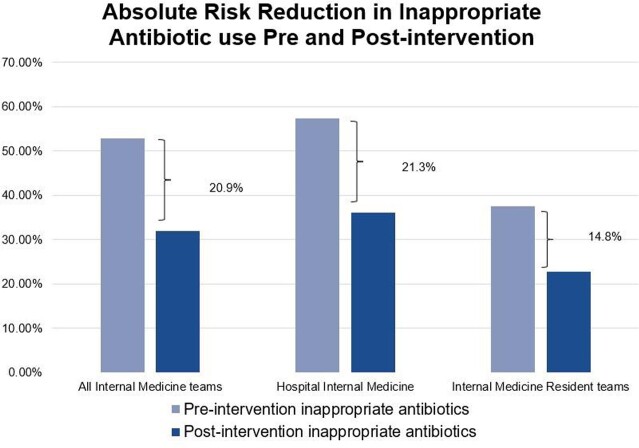

**Conclusion:**

Education on SSTI treatment coupled with use of the MRSA nares PCR appeared to improve empiric antibiotic choices. Residents stated that the nasal MRSA PCR facilitated empiric antibiotic choice. Positive ARR was seen in whole and sub-group analyses of physician teams. We theorize that the lack of statistical significance in the IMR group was related to a small sample size.

**Disclosures:**

**All Authors**: No reported disclosures.

